# Effect of Cervus and Cucumis Peptides on Osteoblast Activity and Fracture Healing in Osteoporotic Bone

**DOI:** 10.1155/2014/958908

**Published:** 2014-11-30

**Authors:** Ai-Yuan Wang, Yue Tian, Mei Yuan, Li Zhang, Ji-Feng Chen, Wen-Jing Xu, Hao-Ye Meng, Xiao-Ming Yu, Yao-Qin Wang, Quan-Yi Guo, Shi-Bi Lu, Jiang Peng, Yu Wang

**Affiliations:** Institute of Orthopedics, Chinese PLA General Hospital, Beijing 100853, China

## Abstract

Osteoporosis is associated with delayed and/or reduced fracture healing. As cervus and cucumis are the traditional Chinese treatments for rheumatoid arthritis, we investigated the effect of supplementation of these peptides (CCP) on bone fracture healing in ovariectomized (OVX) osteoporotic rats *in vitro* and *in vivo*. CCP enhanced osteoblast proliferation and increased alkaline phosphatase activity, matrix mineralization, and expression of runt-related transcription factor 2 (Runx2), bone morphogenetic protein 4 (BMP4), and osteopontin. *In vivo*, female Sprague-Dawley rats underwent ovariectomy and the right femora were fractured and fixed by intramedullary nailing 3 months later. Rats received intraperitoneal injections of either CCP (1.67 mg/kg) or physiological saline every day for 30 days. Fracture healing and callus formation were evaluated by radiography, micro-CT, biomechanical testing, and histology. At 12 weeks after fracture, calluses in CCP-treated bones showed significantly higher torsional strength and greater stiffness than control-treated bones. Bones in CCP-treated rats reunified and were thoroughly remodeled, while two saline-treated rats showed no bone union and incomplete remodeling. Taken together, these results indicate that use of CCP after fracture in osteoporotic rats accelerates mineralization and osteogenesis and improves fracture healing.

## 1. Introduction

Osteoporosis is characterized by low bone mass and microarchitectural deterioration of bony tissue, which leads to bone fragility and increased risk of fracture [1] of the hip, spine, and wrist because of brittle bones. Osteoporosis affects more than 75% of the population in Europe, the United States, and Japan [2] and has been predicted to cause more than 50% of hip fractures in Asia by the year 2050 [3]. According to the US National Institutes of Health, an estimated 10 million Americans have osteoporosis and a further 18 million have low bone mass, placing them at increased risk for the disease. Osteoporosis is responsible for more than 1.5 million fractures annually, including 300,000 hip fractures, 700,000 spinal fractures, 250,000 wrist fractures, and more than 300,000 fractures at other sites on the body [4, 5].

Osteoporosis also impairs healing of fractures. While osteoporosis therapies are designed to boost bone mass and prevent further bone loss, the most common therapy, bisphosphonates (BPs), may have negative effects on healing; animal and clinical studies have found that BPs delay fracture union [6–8].

At present, no pharmacologic treatments are available to enhance fracture healing in osteoporosis [9]. Practitioners of Chinese medicine treat osteoporosis with a combination of mineral-containing herbs, as well as cervus and cucumis peptide (CCP), the combined extracts from deer horn and sweet melon seeds, which has gained popularity in orthopedic clinics in China. CCP is used to promote fracture healing and treat osteoarthritis and rheumatoid arthritis. Clinical observations suggest that CCP use is associated with more rapid bone healing and return to function in some orthopedic conditions [10–16]. In addition to evaluation in clinical trials for efficacy and safety, CCP's bioactive properties, mechanism, and effects on bone fracture in osteoporosis have been investigated.

Here, we investigated the effects of supplementation with CCP extract on bone fracture healing in ovariectomized (OVX) osteoporotic rats* in vivo* and* in vitro*.

## 2. Materials and Methods

### 2.1. Reagents and Peptides

Samples of CCP extract, as well as individual cervus (CHDP) and cucumis (CSMP) peptide extracts, were obtained from Yuheng Pharmaceutical Co. (Harbin, China). Cervus is a seven-amino acid peptide extracted from deer horn, and cucumis is a basic, nine-amino acid linear peptide extracted from the seeds of sweet melon. The average content of amino acids in CCP extract was (in *μ*g/mL): asp 30.75, glu 34.61, ser 8.1, gly 17.14, his 2.64, arg 18.47, thr 5.41, ala 32.38, pro 7.92, tyr 9.74, val 11.93, met 2.84, cys 4.23, ile 8.31, leu 10.12, and phe 9.09. The total content of free amino acids was between 200 and 400 *μ*g/mL. CCP also includes a small amount of calcium (2.20 *μ*g/mL), magnesium (38.0 *μ*g/mL), iron (0.75 *μ*g/mL), and aluminum (0.22 *μ*g/mL) and a large amount of sodium [17]. CCP, CHDP, and CSMP were injected at a concentration of 2 mg/mL.

### 2.2. Cell Lines

Human C2C12 myoblasts, mouse MC3T3E1 osteoblasts, and mouse RAW264.3 monocytes (precursors of osteoclast) were obtained from the cell bank of XieHe Medical University and cultured in Dulbecco's modified Eagle's medium (DMEM; Sigma and Gibco USA).

### 2.3. Cell Culture

Primary osteoblast cells and osteoclast cells were cultured in sterile DMEM (Gibco BRL, Grand Island, NY) supplemented with penicillin (100 U/mL), streptomycin (100 *μ*g/mL), and 10% fetal bovine serum (Yuan Heng Biotechnology Co, Beijing). Cells were grown under standard growth conditions, at 37°C in a humidified atmosphere with 5% CO_2_. When cells reached confluence, they were detached with 0.25% trypsin-0.2% EDTA in phosphate-buffered saline (PBS) and then subcultured after 1 : 2 dilution in a 12.5-cm^2^ volume tissue culture flask. The medium was changed three times per week.

### 2.4. Cell Proliferation

The effect of CCP, CHDP, or CSMP on osteoblast proliferation was analyzed by CCK-8 assay (Dojindo Molecular Technology). Briefly, human C2C12 cells were cultured in 96-well plates (2000 cells/well) and treated with CCP, CHDP, or CSMP (400, 200, or 20 *μ*g/mL), with BMP-2 (500 ng/mL) as a positive control, and allowed to proliferate for 1, 3, 5, 7, or 9 days. MC3T3E1 cells were cultured as above, treated with CCP (100, 10 or 1 *μ*g/mL), and allowed to proliferate for 1, 2, or 3 days.

#### 2.4.1. Bone Formation: Alkaline Phosphatase (AP) Activity and Matrix Mineralization

MC3T3E1 cells were seeded in 24-well plates at 2 × 10^5^/well and treated with CCP (1, 10, and 100 *μ*g/mL), with BMP-2 (250 ng/mL) as a positive control. After 7 days, cells were harvested, rinsed three times with PBS, and lysed with lysis buffer containing Tris, EDTA, and Triton X-100. AP activity of lysate was estimated using the A059 alkaline phosphatase assay kit (Nanjing Jiancheng Bioengineering Co., China) directly from plates. Optical density at 405 nm was read on a Titertek Multiscan absorbance photometer and was corrected for total cellular protein in the same samples as quantified by the Bradford method (Bio-Rad, Inc., Richmond, CA).

For matrix mineralization, MC3T3E1 cells were seeded in six-well plates at 3 × 10^5^/mL/well and treated with CCP (1, 10, and 100 *μ*g/mL). After 14 days, mineralization was assessed by alizarin red staining and quantified according to the method of Schenk et al. [18] as modified by Gregory et al. [19].

### 2.5. Osteoblast Gene Expression

MC3T3E1 cells were seeded in six-well plates at 3 × 10^5^/mL/well, treated with CCP (1, 10, and 100 *μ*g/mL = L), and allowed to proliferate for 3 days. Total RNA (20 pellets per experimental condition and time) was isolated as described [20] and 1 mg RNA was reverse-transcribed using the iScript cDNA Synthesis kit (Toyobo, Japan). Gene expression was normalized to that of the housekeeping gene glyceraldehyde-3-phosphate dehydrogenase (GAPDH). Real-time RT-PCR employed 1 mL of each cDNA sample in triplicate, amplified using an i-Cycler PCR system (Bio-Rad) and the SYBR green PCR Core Kit (Applied Biosystems, Foster City, CA). The primer sequences were osteopontin (OPN), 5′-CTCGATGTCATCCCTGTTGC-3′, 5′-TGCCCTTTCCGTTGTTGTC-3′; runt-related transcription factor 2 (RUNX-2), 5′-GTTCAACGATCTGAGATTTGTG-3′, 5′-GGGAGGATTTGTGAAGACTG-3′; and BMP-4, 5′-GCGGGACTTCGAGGCGACAC-3′, 5′-ATCCGGGATGACGGCGCTCT-3′.

### 2.6. Ovariectomized Rat and Fracture Model

#### 2.6.1. Animals and Treatment

We housed 40 female 4-month-old Wistar rats (300 ± 20 g) for 7 months under proper conditions with an appropriate diet. Permission for the experiment was given by the Ethics Committee of the Hospital of PLA. All rats were ovariectomized (OVX), and osteoporosis was allowed to develop for 3 months, during which rats were fed a diet containing 1.66% calcium and 1.24% phosphate. Following confirmation of osteoporosis by micro-CT, unilateral cross-femoral fractures, at the middiaphyseal level of the right femur, were induced under anesthesia and fixed by intramedullary nailing (1 and 1.2 mm, http://huajiehao.com/, Beijing). OVX rats (*n* = 20 per group) then received intraperitoneal injections of either CCP (1.67 mg/kg) or the same volume of saline beginning on postoperative day 1 and then every day for 30 days. Femora were examined by micro-CT at 4, 6, 8, and 12 weeks after fracture and fixation.

### 2.7. Micro-CT and Image Segmentation

Rat femora were examined by 3D micro-CT (GE eXplore Locus micro-CT system for small animals, RS-9, GE Healthcare, Ontario, Canada) under anesthesia with 10% chloral hydrate (0.3 mL/100 g). Femora were scanned at an isotropic voxel size of 0.046 mm. A fixed length of bone of 100 image layers, located between the proximal and distal boundaries of the callus, was analyzed. The outer boundary of the callus and the area enclosed by the periosteal surface of the preexisting cortical bone were defined by automated segmentation; the volume enclosed by these two surfaces was the callus volume of interest. The MicroView software (GE Healthcare, Ontario, Canada) was used to reconstruct and process the 3D images and calculate the following bone morphometric parameters: total callus volume (TCV) and tissue mineralized density (TMD) [21]. Mineralized tissue was distinguished from unmineralized and poorly mineralized tissue using a fixed global threshold of 25% of the maximum gray value, corresponding to a mineral density of 641.9 mg HA/cm^3^ and approximately 45% of the attenuation of mature cortical bone in the specimens examined. This value was based on visual inspection of the tomograms and qualitative comparison with paired, decalcified histological sections from additional specimens.

### 2.8. Biomechanical Examination

After micro-CT scanning, at 12 weeks, rats were killed and bone and hardware were isolated from the surrounding soft tissue. The proximal and distal ends of the femora of five specimens from each group were embedded in polymethyl methacrylate and mounted on a combined-axial-motion and torsional-testing jig attached to a universal testing machine (858 Mini Bionix II, MTS System Corp., Eden Prairie, MN, USA). The distal end of the specimen was rotated laterally at 6°/min until bone failure was observed. Peak torsion (Nm), failure angle (radians), energy to failure (Nm*·*radian), and torsional stiffness (Nm/°) were calculated from the load displacement curves.

### 2.9. Histological Analysis

Ten femora from each group were analyzed histologically. After the animals were euthanized at 12 weeks, specimens were fixed in 4% formaldehyde at 4°C, decalcified in 15% EDTA for 2 weeks, dehydrated, and paraffin-embedded. Sections (5 *μ*m) were stained with hematoxylin and eosin and examined by microscopy.

### 2.10. Statistical Analysis

Differences were analyzed using SPSS 12.0 (SPSS Inc., Chicago, IL). Data are expressed as means ± SD.* In vitro* results, cell proliferation, ALP activity, and gene expression variables were evaluated using one-way ANOVA.* In vivo* results were compared between groups using Student's *t*-test. A value of *P* < 0.05 was considered to indicate statistical significance.

## 3. Results

### 3.1. CCP Promotes Proliferation and Mineralization of Human Osteoblasts

**Figure 1:**
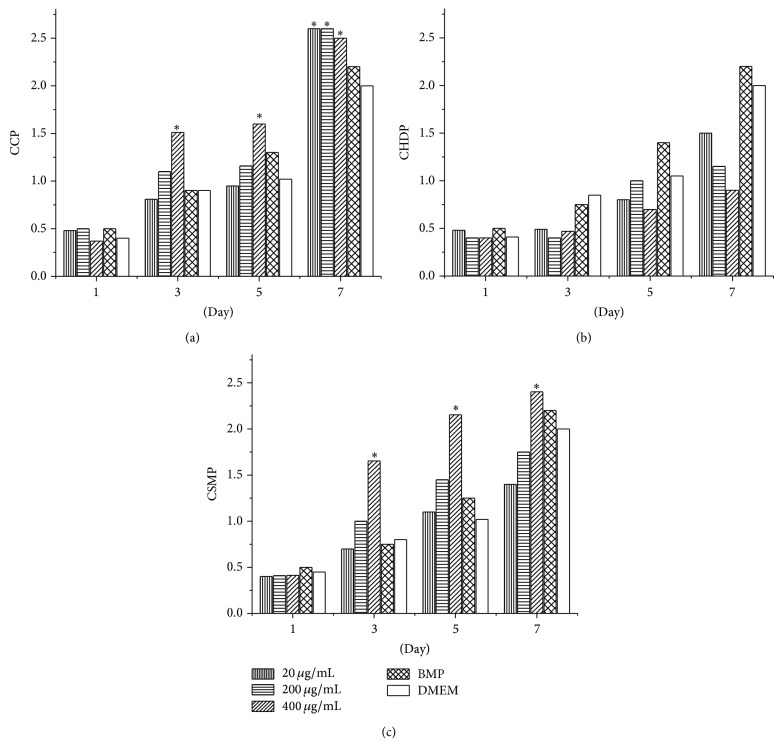
Effect of combined cervus and cucumis peptide (CCP) extracts, cervus peptide extract from deer horn (CHDP), and cucumis extract from sweet melon (CSMP) (dose range 20–400 *μ*g/mL) on proliferation of human osteoblast C2C12 cells. Data are means ± SD of six replicates. ^*^ *P* < 0.01 compared with DMEM.

**Figure 2:**
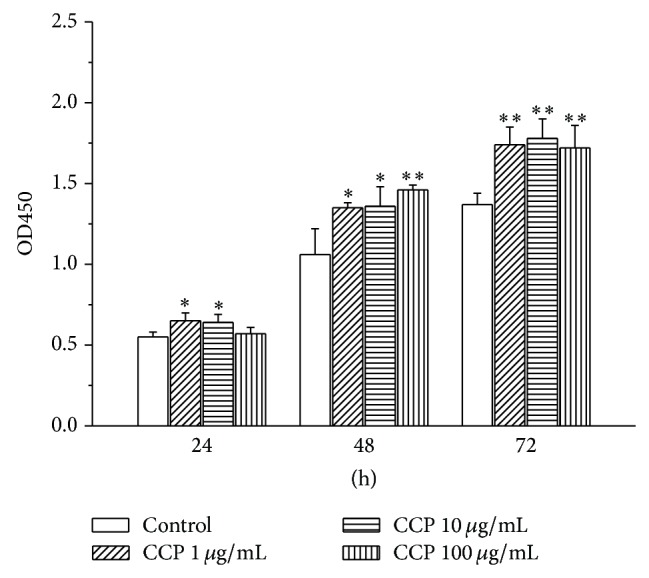
Effect of CCP (dose range 1–100 *μ*g/mL) on proliferation of mouse MC3T3E1 osteoblasts. Data are means ± SD of six replicates. ^*^ *P* < 0.05, ^**^ *P* < 0.01 compared with the control.

The effect of CCP on human C2C12 osteoblast proliferation was analyzed by CCK-8 assay. The growth rate of C2C12 cells was enhanced by all concentrations of CCP and was higher than that following treatment with BMP (*P* < 0.05) (Figure 1). C2C12 proliferation was also enhanced in a dose-dependent manner by CSMP, and the highest dose (400 *μ*g/mL) was more effective in stimulating proliferation than BMP (*P* < 0.05). All concentrations of CHDP had no significant effect on proliferation. CCP also enhanced the proliferation of mouse MC3T3E1 osteoblasts in a dose- and time-dependent manner (48 h, *P* < 0.05; 72 h, *P* < 0.01) (Figure 2).

**Figure 3:**
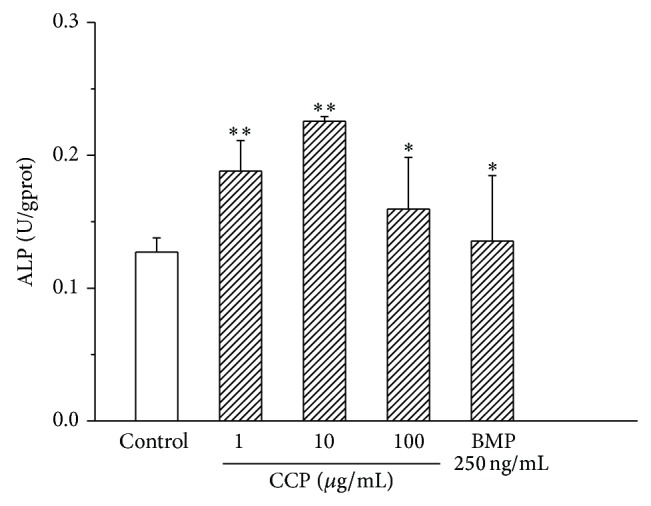
Effect of CCP on alkaline phosphatase (ALP) activity in mouse MC3T3E1 osteoblasts. Data are means ± SD of six replicates. ^**^ *P* < 0.01, ^*^ *P* < 0.05 versus control.

**Figure 4:**
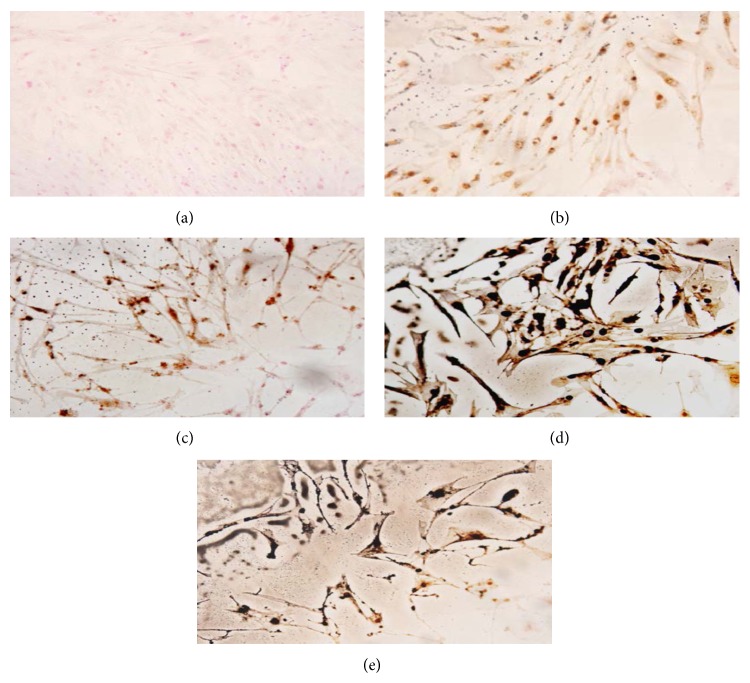
Effect of saline (a), CHDP (b), CSMP (c), BMP (d), and CCP (e) on matrix mineralization by human C2C12 osteoblasts (Von Kossa staining).

**Figure 5:**
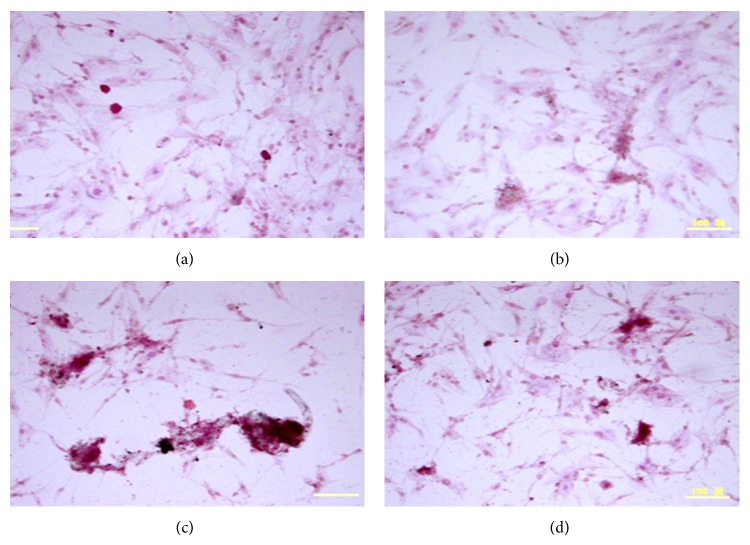
Effect of CCP on matrix mineralization by mouse MC3T3E1 osteoblasts (alizarin red staining). (a) Control; (b), (c), and (d) 1, 10, and 100 *μ*g/mL CCP.

We also examined the effect of CCP on AP activity and mineralization. CCP treatment increased AP activity in MC3T3E1 cells relative to control treatment; 1 and 10 *μ*g/mL treatments caused significant effects (*P* < 0.01 and *P* < 0.05) (Figure 3). CCP, CSMP, and CHDP also enhanced mineralization in these cells (Figure 4). However, in human osteoblasts, the effect of CCP on mineralization was not dose-dependent; the 10 *μ*g/mL treatment (among 1, 10, and 100 *μ*g/mL) caused the greatest effect (Figure 5).

### 3.2. CCP Increases mRNA Expression of Bone Markers

**Figure 6:**
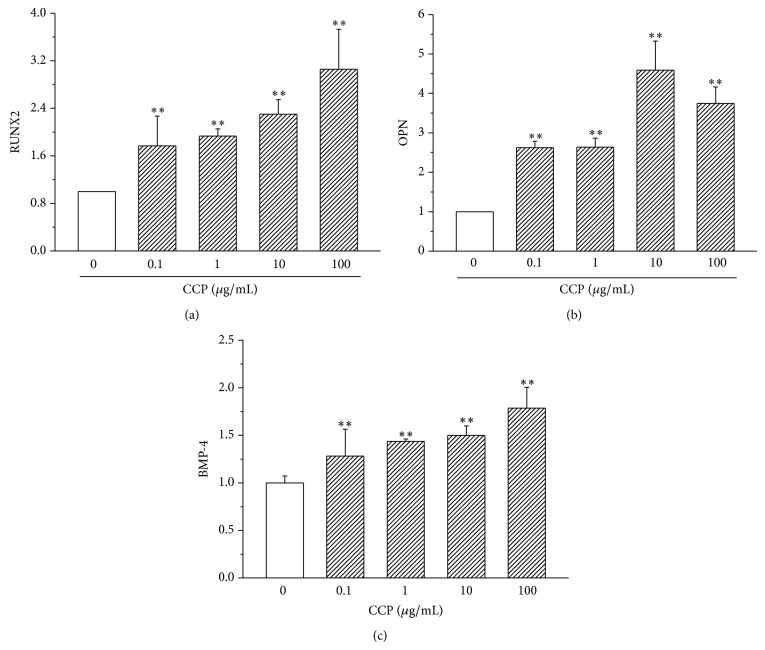
Effect of CCP on expression of RUNX2, OPN, and BMP-4 in MC3T3E1 osteoblasts (quantitative RT-PCR). ^**^ *P* < 0.01.

CCP significantly increased expression of BMP-4, RUNX-2, and OPN in MC3T3E1 cells. Effects on expression of RUNX and BMP-4 were greatest at 100 *μ*g/mL CCP, while those on expression of OPN were maximal at 10 *μ*g/mL (Figure 6).

### 3.3. Effect of CCP on Femoral Fracture Repair in Osteoporosis

#### 3.3.1. Micro-CT Confirmation of Osteoporosis Induction

**Figure 7:**
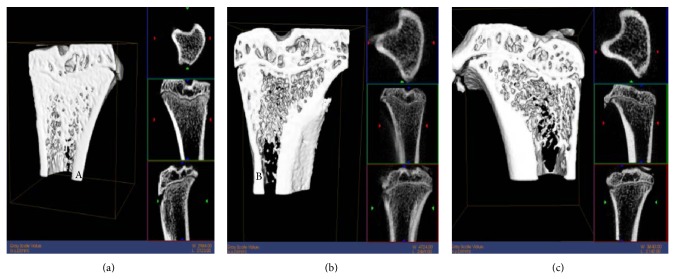
Micro-CT of femurs in rats with ovariectomy-induced osteoporosis. (a) Normal bone; (b) 2 weeks and (c) 4 weeks after ovariectomy.

Osteoporosis was induced in rats by ovariectomy (OVX). Micro-CT of the proximal tibia at 3 months after OVX revealed decreased bone mineral density, trabecular bone (the core component, essential for bone strength), bone mineral density (BMD), volume fraction of bone (BVF), and trabecular connections and increased trabecular space (Tb.Sp) (Table 1 and Figure 7).

#### 3.3.2. Radiographic Analysis

**Figure 8:**
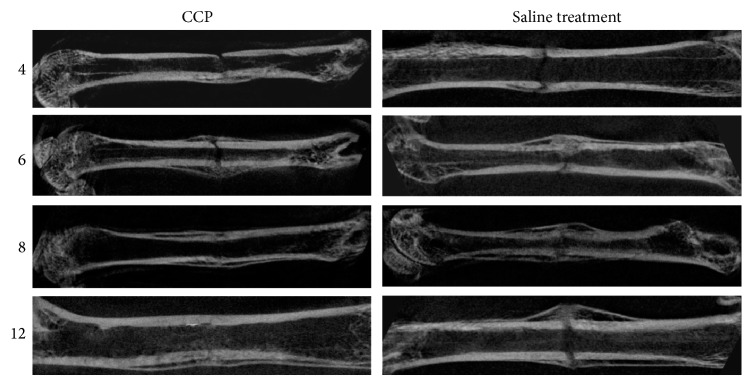
Micro-CT examination of the effect of CCP treatment (intraperitoneal injection, 1.67 mg/kg, beginning on postoperative day 1 and then every day for 30 days) on healing of femoral fractures at 4, 6, 8, and 12 weeks.

We evaluated the effects of CCP on callus formation, remodeling, and bone union at weeks 4, 6, 8, and 12 after treatment. Callus formation and bone union increased every week with CCP treatment; bone union and remodeling were observed in all 10 treated rats at 12 weeks (Figure 8). In control rats, a large callus was formed, and remodeling remained incomplete in 9 of 10 rats, with nonunion in 2 rats at up to 12 weeks (Figure 8).

#### 3.3.3. Micro-CT Analysis

A callus was formed in both groups at 2 weeks; its volume increased up to 6 weeks and decreased thereafter in both groups (Table 2). BMD of the newly formed callus gradually increased in each group. With CCP treatment, BMD reached a maximum at 6 weeks (*P* < 0.05 versus 4 weeks), while in control rats BMD remained stable through week 6; the increase only reached significance at 12 weeks (*P* < 0.01). Thus, CCP accelerated bone mineralization following fracture.

#### 3.3.4. Biomechanical Examination

We assessed the strength of healed bone by biomechanical testing at 12 weeks after fracture and treatment. Five CCP-treated and control femurs were cleaned of soft tissue and stored at −20°C; contralateral femurs served as standards of normal bone strength. Peak torsion, energy absorption, stiffness, and load were greater following CCP treatment than in control femurs (*P* < 0.01), with no difference in torsional stiffness (*P* > 0.05) (Table 3).

#### 3.3.5. Histopathology

**Figure 9:**
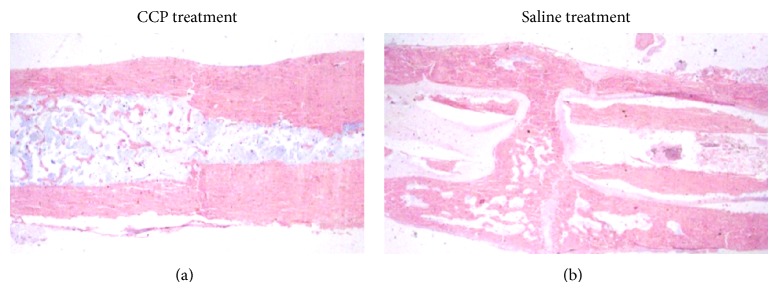
Histological examination by H&E staining of the effect of CCP treatment (intraperitoneal injection, 1.67 mg/kg, beginning on postoperative day 1 and then every day for 30 days) on healing of femoral fractures at 12 weeks.

At 12 weeks after fracture, the fracture gap was bridged in CCP-treated femurs, with complete mature bony continuity and complete remodeling in all six specimens (Figure 9). Following control treatment, the fracture area was surrounded by newly formed callus, and bone remodeling was not complete in three of six specimens; in one case, the bone gap was not bridged and the callus contained focal defects.

## 4. Discussion

Osteoporosis, which results from an imbalance between the rates of bone formation and bone resorption, increases the risk of fracture by decreasing bone mineral density, disrupting bone microarchitecture, and reducing levels of noncollagenous proteins. Osteoporotic fracture is the most common complication of osteoporosis, which occurs with minimal trauma because the bone is so fragile. Fragility fractures heal despite the abnormal bone remodeling in osteoporosis, but the effect of osteoporosis on the complex process of fracture healing in humans is not well understood. However, some laboratory studies have shown that osteoporosis impairs fracture healing in both the early and late period [22–25].

Here, we investigated the effects of supplementation with CCP extract, a traditional Chinese medicine, on bone fracture healing in OVX osteoporotic rats* in vivo* and* in vitro*. At 12 weeks after surgical fracture, calluses in CCP-treated bones had significantly higher torsional strength and greater stiffness than with control treatment. Fracture healing in all CCP-treated rats involved union and remodeling.* In vitro*, CCP enhanced osteoblast cell proliferation and increased AP activity, matrix mineralization, and expression of RUNX-2, BMP-4, and OPN. Postfracture use of CCP in osteoporotic rats accelerated mineralization and osteogenesis and was associated with improved fracture healing.

Fracture healing at early stages in osteoporotic rats was poor. Even at 12 weeks after fracture, healing remained incomplete; calluses' strain and load parameters remained lower than those of intact bone. Namkung-Matthai et al. observed a 40% reduction in callus cross-sectional area in osteoporotic rats [22] and Walsh et al. found decreased mechanical strength, which slows fracture healing [23]. Similarly, Xu et al. and Kubo et al. showed that osteoporosis impairs the early and the late periods of fracture healing, respectively [24, 25].

In our study, we first examined whether CCP treatment enhances fracture healing in the OVX rat model of osteoporosis. Healing was assessed by micro-CT, radiography, and biomechanical strength measurements at the fracture site, as well as histology. CCP treatment accelerated mineralization and fracture healing and shortened remodeling time as compared with control treatment. This peptide combination also improved the biomechanical properties of the fractured callus, increasing the stiffness and load of fractured bone in torsional testing, at a late period of healing. Overall, CCP had a positive effect on fracture healing, in agreement with previous clinical results [13].

However, the mechanism by which CCP promotes bone fracture healing remains unclear. In the present study, we first showed that CCP extracted from deer horn and sweet melon seeds significantly enhanced osteoblast proliferation and differentiation, as indicated by increased mineralization and AP activity and increased expression of OPN, BMP-4, and RUNX-2* in vitro*. The latter results confirm those of Duan et al. that CCP increases BMP-2 expression in osteoblasts [20] and Xu et al. who reported increased RUNX expression in osteoblasts following CCP treatment [26].

We also examined cytokine expression in the osteoclast precursor cell line RAW264.3 and found that CSMP increased vascular endothelial growth factor secretion in osteoclast precursor cells (data not shown), which suggests that the peptide would induce blood vessel formation and enhance bone repair* in vivo*. This result is in agreement with the findings of Wang et al. [27]. We did not examine whether CCP influences early fracture healing. Ju et al. reported that CCP decreases expression of tumor necrosis factor *α*, interleukin 5, and tissue inhibitor of matrix metalloproteinase 1 in the synovial membrane and serum of rats [28, 29], which may decrease inflammation shortly after fracture. CCP may enhance secretion of growth factors by osteoblasts shortly after fracture, but further study is needed to confirm this inference.

To analyze which component is responsible for the activity of CCP, we compared the effects of either CHDP or CSMP on the proliferation of human C2C12 osteoblasts. The highest dose (400 *μ*g/mL) of CSMP had an identical effect on proliferation as CCP, and the smaller doses (200 and 20 *μ*g/mL) of CSMP produced lesser effects than CCP. The highest dose of CHDP (400 *μ*g/mL) did not significantly enhance proliferation of human osteoblasts at the early phase (1–7 days). CHDP at a low dose (20 *μ*g/mL) produced effects identical to the high dose of CCP (Figure 1).

In our study, both high and low doses of CCP (1, 10, and 100 *μ*g/mL) significantly enhanced mouse MC3T3C1 osteoblast proliferation* in vitro*. However, for* in vivo* experiments, only one dose was employed (1.67 mg/kg) based on the literature [20, 27, 28]. Determining the optimum dose for clinical trials requires further study.

High expression of Runx-2 and OPN indicate osteoblast maturation and differentiation [30]. In the current study, CCP enhanced expression of both osteoblasts, suggesting that CCP promotes osteoblast differentiation and extracellular matrix mineralization, thereby enhancing bone fracture healing. However, whether CCP extract contains cytokines responsible for these effects requires further investigation.

## 5. Conclusions

CCP enhances osteoblast proliferation and increases matrix mineralization and expression of RUNX2, OPN, and BMP4. Postfracture administration of CCP in osteoporotic rats accelerates mineralization and osteogenesis and is associated with improved fracture healing.

## Figures and Tables

**Table 1:** Micro-CT-derived bone structure indices in ovariectomized rats.

Week	BMD (mg/cm)	BVF (mg/cc)	Tb.Sp (mm)	Structure of model index
0	237.70 ± 57.00	0.45 ± 0.22	0.24 ± 0.15	−1.27 ± 6.27
4	133.90 ± 58.72	0.27 ± 0.11	0.44 ± 0.30	1.48 ± 0.22
8	97.25 ± 32.36	0.19 ± 0.06	0.64 ± 0.25	1.62 ± 0.23
12	73.53 ± 26.21	0.15 ± 0.05	0.82 ± 0.27	1.60 ± 1.14

BMD: bone mineral density; BVF: volume fraction of bone; Tb.Sp: trabecular space; structure of model index and ratio of plates to rods in cancellous bone, where 0 indicates all plates and 3 indicates all rods.

**Table 2:** Micro-CT-derived fracture repair indices in cervus and cucumis polypeptide- (CCP-) treated rats.

Week	CCP treatment		Saline treatment	
	Total callus volume (mm^3^)	BMD (mg/cm)	Total callus volume (mm^3^)	BMD (mg/cm)
4	83.18 ± 20.69	661.59 ± 58.54	82.56 ± 18.25	668.96 ± 52.31
6	91.46 ± 20.02	736.45 ± 45.54^a,b^	97.49 ± 32.61	666.67 ± 41.12^a^
8	72.43 ± 11.10	788.35 ± 48.95^b^	80.28 ± 20.80	742.01 ± 92.51
12	74.87 ± 34.54	801.05 ± 60.86^b^	70.78 ± 14.21	770.05 ± 42.35^b^

^a^ *P* < 0.05 compared to control; ^b^ *P* < 0.05 compared to at 4 weeks.

**Table 3:** Biomechanical indices of postfracture bone strength in CCP-treated rats.

Section	Diameter (mm)	Peak torsion (Nm)	Torsional stiffness (Nm/deg)	Energy absorption
Native	3.75 ± 0.28	0.41 ± 0.11	1.68 ± 0.71	0.11 ± 0.04
Control	4.19 ± 1.01	0.27 ± 0.05	0.98 ± 0.65	0.04 ± 0.00
CCP	4.50 ± 0.83	0.41 ± 0.08^**^	1.26 ± 0.49	1.14 ± 0.02^**^

CCP treatment compared to OVX control: ^**^ *P* < 0.01.
